# Male and female Asians living with HIV: a text-mining analysis comparing their concerns

**DOI:** 10.3389/fpsyg.2023.1114731

**Published:** 2023-05-12

**Authors:** Wei-Ti Chen, Russell Barbour

**Affiliations:** ^1^School of Nursing, University of California, Los Angeles, Los Angeles, CA, United States; ^2^Center for Interdisciplinary Research on AIDS (CIRA), Yale School of Public Health, New Haven, CT, United States

**Keywords:** text mining, HIV, gender, antiretroviral therapy, concerns, Asians

## Abstract

**Introduction:**

Asians are “a forgotten population” whose HIV prevention and treatment needs have long been ignored. Studies on people living with HIV/AIDS (PLWHA) have primarily reported on physical and psychological conditions among men and gender minorities.

**Methods:**

We used data-mining to select words and word patterns from pooled in-depth interviews conducted with 33 women and 12 men (*n* = 45) who were HIV positive and residing in San Francisco, United States, Shanghai, Beijing, or Taipei, China. We analyzed gender-based data (male vs. female) within the participant responses.

**Results:**

Disclosure of HIV serostatus was discussed by both genders of PLWHA. Participants were concerned whether they should disclose their diagnosis and how to disclose it to their family members. Specifically, for women, family relationships and financial burdens were discussed most often. In terms of men, HIV disclosure was the key concern followed by disclosure of their sexual preference and concerns over what people were saying about them in the community.

**Conclusion:**

This project compared and contrasted concerns of Asian HIV-positive men and women. As healthcare providers promoting self-management by HIV-positive individuals of both genders, it is important to consider that there may be significant differences between them. Future interventions should consider how gender role influences self-management strategies in PLWHA and how support can be targeted to achieve a better quality of life for PLWHA.

## 1. Introduction

Since the advent of antiretroviral therapy (ART), HIV has been considered a chronic but manageable condition. By lowering viral loads, ART reduces the risk of HIV transmission from infected to uninfected individuals. However, living with the symptoms of HIV, side effects from ART, medication adherence, health-related costs, tasks associated with self-management of the disease, and potential medication resistance, can all add stress to the lives of the HIV-positive individuals (Aantjes et al., 2014).

Studies have found that people living with HIV/AIDS (PLWHA) often have to manage multiple conditions and are still highly discriminated against in society (Paudel and Baral, 2015). Depression is common in PLWHA, especially for those experiencing social isolation or who have been expelled from their families (Lanier and DeMarco, 2015). Loss of friends or loved ones, worries about children, experience of stigma, and substance use can further exacerbate depression (Parcesepe et al., 2019). Sleep disturbance, poor adherence to ART, poor hygiene, forgetfulness, weight loss or gain, and overt sadness are all possible signs of depression (Chen et al., 2013). HIV-related stigma has been reported differently in men and women and how they cope with stigma are divergent (Parcesepe et al., 2019). In addition, perception of quality of life are also different between genders (Vo et al., 2016). However, limited studies have addressed the question of whether there are differences in concerns between HIV-positive men and women as well as the potential factors that drive these crucial differences.

Comparative gender analyses have shown that pain tolerance, discomfort patterns, and mental stress are generally experienced differently between men and women with chronic disease (Hamilton et al., 2022). Women are twice as likely as men to experience depression and this increased risk is likely related to gender influences (Eid et al., 2019). However, studies on PLWHA have primarily reported on physical and psychological conditions among men and gender minorities (Rooney et al., 2018). Therefore, this study examined gender differences in the concerns of HIV-positive Asian populations.

Asian Americans are “a forgotten population” whose HIV prevention and treatment needs have long been ignored (Li et al., 2017). The percentage of Asian American PLWHA has drastically increased from less than 1% of the total HIV population in 2005 to more than 2% in 2014, HIV Among Asian Americans (2020) with a within-group increase of nearly 70% (HIV Among Asian Americans, 2020). As Asian Americans have not been a traditional target of vigorous prevention and research efforts, many Asian American communities have yet to be well prepared to address HIV and related issues. Therefore, our understanding of Asian American PLWHA regarding their self-management and medical adherence remains limited. The little available literature indicates that Asian American PLWHA share some similar concerns about HIV and treatment with other PLWHA of other ethnicities. These shared concerns include management of HIV-induced symptoms, side effects of ART, and quality of life (Chen et al., 2018). However, Asian American PLWHA face additional challenges arising from their immigration status and family-centered cultures including concerns over family obligations, HIV stigma, serostatus disclosure, medication access and adherence, and acculturation pressure (HIV Among Asian Americans, 2020).

HIV rates are slowly increasing in Asian people (HIV Surveillance Annual Report, 2014; UNAIDS, 2015) These PLWHA report poor quality of life and suffer from deteriorating physical and mental health (Yang et al., 2016). In this study, based on a previously collected dataset from 2006 to 2013 Chen et al. (2018, 2019) we analyzed 45 in-depth interviews among Asians PLWHA from Beijing, Shanghai, Taipei, and San Francisco.

## 2. Participants, ethics, and methods

### 2.1. Ethics statement

The study was approved by local ethical committees and written informed consent was obtained from all participants.

### 2.2. Study design and participants

The study aimed to identify if there were any gender-based differences (men vs. women) in the concerns reported by HIV-positive Asian populations. We analyzed data collected in San Francisco, Shanghai, Beijing, and Taipei by using data mining techniques to expose concealed “messages” that were present in the qualitative dataset (Reardon, 2014). Although the Asians in the study came from diverse backgrounds, the study participants observed similar cultural norms and held similar family values in dealing with chronic disease (Asian and Pacific Islander Wellness Center, 2013).

#### 2.2.1. Participants

The preexisting, qualitative dataset gathered from 33 HIV-positive women and 12 HIV-positive men (*n* = 45) that included HIV-positive Chinese, Taiwanese, and Asian Americans in Shanghai (*n* = 16), Beijing (*n* = 10), Taipei (*n* = 3), and San Francisco (*n* = 16) was re-analyzed. Study participants needed to be at least 18 years old, have no cognitive impairments, be confirmed HIV-positive, and able to spend 1 h with researchers for an in-depth interview. Gender was self-identified as female or male and included transgender participants. Participants’ ages ranged from 25 to 69 years old, with a mean of 44 years. Place of origin was reported as Indonesia (1), Japan (1), Philippines (6), Malaysia (1), Cambodia (1), Vietnam (4), Native Hawaii (1), Taiwan (4), and China (26).

### 2.3. Data collection

The relevant institutional review boards reviewed and approved the study. All methods were performed in accordance of human subject protections guidelines and regulations (e.g., Declaration of Helsinki) and all study protocols were approved by the involved institutions. Informed consent was obtained from each study participant. Experienced qualitative research staff conducted in-depth interviews with participants. A semi-structured interview checklist was used to make sure all the essential questions and topics were covered. Specifically, information about lived experiences with HIV was obtained in detail. Some of the sample questions were: “How has your life been since you learned you had HIV?”; “How do you manage your disease?”; and “What are your concerns with HIV?.” Participant interviews were audio-recorded with permission and transcribed. For those participants who declined to be recorded, detailed notes were made of their responses for later analysis.

In this project, four study sites were involved: San Francisco (*N* = 15), Shanghai (*N =* 19), Beijing (*N =* 7) and Taipei (*N =* 4). Table 1 presents the year data was collected, its location, and number of study participants enrolled from each study site. Snowball sampling was employed to recruit potential study participants. Study collaborators and providers at infectious disease hospitals and clinical centers distributed information regarding the research studies. On-site research staff talked to interested participants about the study and answered any questions, obtained study consent, and then scheduled in-depth interviews with the study participants.

**Table 1:** Number of participants by study site location and data collection period.

Study site	Year	Participants
Beijing	2007	10
Shanghai	2010	16
Taipei	2012	3
San Francisco	2013	16

All in-depth interviews were at least 1 h long and were conducted in a private setting. Based on their stated preference, in-depth interviews were conducted with participants in English or Chinese. After each interview done in English, the content was transcribed verbatim. For in-depth interviews conducted in Chinese, transcription was done verbatim in Chinese then translated to English for analysis. In Beijing, bilingual research staff as well as Chinese physicians and nurses conducted the interviews (Simoni et al., 2011). At the rest of the study locations, a research team of bilingual, bicultural Asian Americans conducted the interviews.

### 2.4. Statistical analysis

We used data mining as a method to explore the patterns of gender differences among Asian PLWHA. Data mining analysis is used to capture information from a large body of textual data and can predict whether an emerging field will become a long-lasting source of academic interest or whether it is simply a passing source of interest that will soon disappear (Reardon, 2014). The goal of data mining in qualitative data analysis is to find significant words, phrases, and rules concerning a given research question. The data mining is comprised of three steps: feature extraction (segmenting and categorizing words to use in the next process), the mining process (clustering or associating words by cluster analysis or correspondence analysis), and visualization (creating graphs or tables to visually summarize the data; Ando et al., 2007). In this project, data mining techniques were used to extract meaningful words and word patterns from transcribed in-depth interviews of Asian PLWHA. The data were analyzed using the “***tm*** package in ***R***,” (Feinerer et al., 2008) software that transforms text into workable data. After assembling the data, we categorized concerns by gender according to the top 15 most mentioned concerns for each male (*N* = 12) vs. female (*N* = 33; Feinerer et al., 2008). Other demographic information on the study participants have been published elsewhere (Chen and Barbour, 2017).

There were several processes undertaken in order to prepare the data for the data mining analysis. First, each sentence needed to be parsed; all the punctuation, including quotation marks, question marks, periods, commas, and colons were removed. Second, all the words with similar meanings were merged. For example, “AIDS” was replaced with “HIV” and “discrimination” and “stigma” were merged as “stigma.” Third, numbers and articles (the, a, and an) were deleted, leaving only the meaningful words. The remaining words became the “fragments,” leaving 113 fragments for data mining analysis.

Next, in order to determine the participants’ perception of family support, a dissimilarity analysis was conducted in which we divided all the in-depth interviews by gender (male vs. female). During the in-depth interviews, all study participants were asked to share how their family was involved in their HIV-related care (e.g., taking care of their housework or financial support). According to Feinerer and Hornik (2015), dissimilarity analysis is used to explore the similarity (pair-to-pair) between study groups (in this case, male vs. female). In the ***tm*** package for ***R*** software, male and female quotations were matched via a set of questions and used to calculate the Jaccard coefficient (Feinerer and Hornik, 2015). The Jaccard coefficient expresses the similarity/dissimilarity of the genders on phrases associated with family support. For the qualitative in-depth interviews, the Jaccard formula was set to match the total weight of similar terms to the total weight of terms that were present in either of the two groups (Feinerer and Hornik, 2015).

In the ***tm*** package, a term-document matrix is initially created that includes all words found in the documents (Feinerer and Hornik, 2015). It is assumed that some of the terms (the sparse terms) will be found infrequently or not at all within and across documents, creating empty cells within the matrix (i.e., terms occurring 0 times in a document). A sparse term is defined as giving the minimum percentage of empty cells permitted. Such sparse terms are then removed from the analysis. As recommended in the ***tm*** documentation, Feinerer and Hornik (2015) a sparse factor of 20% was applied in this analysis. Thus, 20% of the terms in the original matrix were removed from the final analysis. Fractional rounding of the sparse factor resulted in removal of 99% of these sparse terms.

## 3. Results

### 3.1. Sample characteristics

Sentence fragments for both males and females that represented typical concerns regarding their lives were selected. Upon eliminating the sparse terms, the software presented the top 1% of the most frequently used words from the 45 study cases that formed the study dataset. The process revealed phrases or words that showed up in the transcripts more frequently than 281 times among the 45 interviews. For all study participants, the highest-ranking fragments in order of frequency were: “care,” “daughter,” “disease,” “family,” “feel,” “HIV,” “hospital,” “husband,” “medicine,” “money,” “son,” “people,” “tell/disclosure,” “thought,” “want,” and “years.” We later identified categories for the top 15 most used words/phrases (see Table 2).

**Table 2:** Sentence fragment frequency by gender.

Male			Female	
Order	Fragment	Frequency	Fragment	Frequency
1	Me	3,217	Hospital	1,257
2	Know	900	Medicine	1,233
3	Time	439	Disease	1,131
4	Want	418	Husband	980
5	HIV	325	Ask	980
6	Feel	265	HIV	909
7	Asian	214	Daughter	762
8	Gay	179	Home	747
9	Years	172	Family	604
10	Told	170	Money	586
11	See	162	Feel	569
12	Back	153	Patients	385
13	Thought	59	Doctors	377
14	I like	34	Afraid	297
15	People said	6	People said	14

Next, we repeated the process but divided the group into males vs. females. First, we tested the most frequent terms within the group of male participants. These terms showed up in the text more than 80 times and included: “Asian,” “back,” “feel,” “gay,” “me,” “know,” “people said,” “HIV,” “see,” “told,” “thought,” “want,” “years,” “time,” and “I like.” Next, we determined the most frequent terms among female participants. These terms showed up more than 200 times and comprised: “afraid,” “ask,” “daughter,” “disease,” “doctors,” “family,” “feel,” “HIV,” “home,” “hospital,” “husband,” “medicine,” “money,” “patients,” and “people said” (see Table 2).

Applying the Theory of Silencing the Self and the Social Ecological Model, Lanier and DeMarco (2015) these terms reflected their individual HIV experinces (e.g., patient, gay, me, afriaid, medicine), living environment (e.g., money, U.S. or in Asia), family and friends’ support system (e.g., people said, daughter, husband, family), and cultural believes (e.g., back home, disease). This process conceptualized how the individual, family circle as well as the community had impacted Asians living with HIV.

## 4. Discussion

This study explores gender differences with regards to concerns of Asians living with HIV/AIDS. The results show that Asian men and women living with HIV/AIDS were concerned with significantly different aspects of their life. Male participants, most of whom were men who have sex with men (MSM), were focused more on their own outcomes. By contrast, HIV-positive women worried more about their family situations.

The topic of disclosure was about equally discussed by both genders of PLWHA and included utterances of “told/disclosure,” “know,” “people said,” and “ask.” Participants were concerned whether they should disclose their diagnosis to their family members. The topic of how to disclose was another common concern for both groups. PLWHA often wonder whether they should disclose partially (e.g., disclose that they have some unspecified blood-related disease) or disclose fully (mention HIV and AIDS) (Simoni et al., 2015). Without disclosing, PLWHA will not be able to get the extra support they need from their families. On the other hand, by disclosing, they may push their family away due to HIV stigma (Chen et al., 2018). As an example, some PLWHA reported that after disclosure they were not invited to family gatherings, e.g., Lunar New Year celebrations and family reunions (Chen et al., 2018).

Telling the truth about their HIV status or discussing the future with children or other family members seemed to be very difficult for the Asian PLWHA in this study. The theme of children was common among both Asian men and women with HIV. The participants often mentioned worrying about their children’s attitudes toward them. Similarly, a past study reported that HIV-positive parents consider the potential stigma from their children and the unknown consequences of disclosure when deciding whether to disclose or not (Chen et al., 2018). Further, articles describe how Asians tend to have strong family values, reflecting a collective culture (Weinstein, 2014; Huang et al., 2021). In this project, women were especially worried about whether their children could still accept them with HIV, figuring heavily into their decision about whether to disclose their status, especially to a daughter whose support they might someday need.

This supports the findings of other studies, done in Asians, that have shown that many PLWHA do not want to become a burden to others (Mao et al., 2018). The decision to not disclose may also be influenced by Confucian principles, which are instilled in many Asians from a young age (Ho and Goh, 2017). These principles hold that the individual should not let their personal misfortunes or unhappiness destroy family harmony (Chen et al., 2018). In order to address this belief, healthcare providers (HCP) should discuss PLWHA’s emotions and how they may adjust their family roles after diagnosis; (Aantjes et al., 2014) thus, PLWHA can prepare for disclosure when and how they choose to. After disclosure, family and social support become key to preventing the situation from getting worse. Among PLWHA, social support is one of the main concerns to accessing other supports (Chen et al., 2018). Asians are expected to stay close to their family members, especially their male descendants (Laidlaw et al., 2010). Yet, an HIV-positive person may be afraid that he or she will transmit the disease to loved ones, highlighting the need for patient education. Therefore, social support should be evaluated during each health care visit, and clinicians should ensure that patients and their families are educated as to the actual transmission routes of the disease (Chen et al., 2018).

For female participants, family relationships and financial burdens were discussed most frequently, which is significant given the fact that middle-aged HIV-positive women need more health care and social service resources as they navigate the health and social-psychological challenges of successful aging (Murphy et al., 2012). Difficulties these women experience include a lack of income and/or health insurance, an inflexible work schedule, disruption of the family relationship, and loneliness (Li et al., 2017). Studies have shown that single HIV-positive women felt that they were no longer “normal” and as a result would not be able to find a partner. At the same time, they felt they could not live alone (Wang et al., 2016). In addition, role expectations and social responsibilities, including caring for family members in the case of many HIV-positive women, remain.

The male participants, who were mostly residing in San Francisco, had different self-perception than the female participants. Some male participants expressed that they saw themselves as openly gay; however, HIV disclosure was a key concern for them and they still worried about what people would say about them in the Asian community. Importantly, studies have reported that many MSM in Asia were married to women as a result of social pressure (Li et al., 2017). We discovered that even male study participants who were living in a liberal society, for example, in MSM-friendly San Francisco, still had concerns about potential disclosure to people in their social circle who lived in Asia. Unlike women in our study, men were concerned about the impact of “double disclosure” of being gay and HIV-positive (Chen et al., 2018). As a result of the increased potential for isolation, males tended to disclose their serostatus very selectively. Most of the time, these Asian men were willing to disclose that they were HIV-positive, but not willing to admit that they may have become infected through a same-sex relationship (Li et al., 2017). Interestingly in this study, all male participants who were recruited in San Francisco typically did not mention anything about family. As immigrants, these men might not have had family members close by and therefore did not see family support as an urgent need. However, as mentioned above, they were still concerned about disclosure and had more self-awareness as they expressed that they preferred to be in charge of their own lives in order to achieve their “American dreams” (Chen et al., 2015).

### 4.1. Limitations

There were limitations in this innovative study. First, the text-mining software ***R*** is an English-only software and therefore we needed to recruit several bilingual research staff to translate the transcriptions for data mining analysis. Translators used in the study varied in their translation styles. Therefore, variations in the frequency of the words/phases is possible. Second, since the in-depth interviews were performed in several different cities in Asia and only one in the United States, the healthcare systems and level of support from the government was different from site to site. However, concerns about HIV were similar among the study participants, regardless of where they lived. Third, this study was a qualitative study design with relatively few participants which limits the ability to generalize the results. Fourth, sample bias could be present as these study participants were not randomly recruited. Fifth, the length of time living with HIV may also have influenced the concerns of these HIV-positive individuals. For example, PLWHA who were diagnosed with HIV within 1 year of being recruited for the study may have been more worried about their physical condition relative to those who had been living with HIV for longer, while those PLWHA who had been diagnosed for a longer period of time may have been more concerned about how to achieve a better quality of life. Therefore, the data might have been skewed. Also, as the data was obtained across a 10-year period, therefore, its findings may not generalize to Asians in current day. Finally, we included more women than men in the study. For future projects, analyzing only males or females may present results that are more broadly applicable.

## 5. Conclusion

Data mining is a relatively new methodology that is available to analyze large quantities of health-related data. By using it in health-related research, we can obtain different perspectives on the populations we are concerned with. For example, with this data, researchers can be more aware of the diverse concerns that different genders have on various health issues. This project compared and contrasted concerns of Asian HIV-positive men and women. As healthcare providers promoting self-management by HIV-positive individuals of both genders, we also need to consider that there may be significant differences between them.

Based on our findings, we would recommend that self-management strategies include a disclosure intervention to enhance social support, particularly for males. Such an intervention should be considered for future study and include a discussion of whether to disclose, when to disclose, how, and to whom. This study reveals that Asian PLWHA worry about the possibility of unwanted or unplanned disclosures of their HIV-positive diagnosis. In some cases, these worries about potential disclosures could contribute to psychological stress or depression. Therefore, we conclude that evaluating a patient’s mental status, particularly with regard to perceived stress, should be a standard procedure for clinicians, at least until there are more studies on structured interventions.

## Data availability statement

The raw data supporting the conclusions of this article will be made available by the authors, without undue reservation.

## Ethics statement

The studies involving human participants were reviewed and approved by the University of Washington and Yale University. The patients/participants provided their written informed consent to participate in this study.

## Author contributions

W-TC: design, recruit, transcribe, translate, analyze and write of the manuscript. RB: analyze and proofread the manuscript. All authors contributed to the article and approved the submitted version.

## Funding

This publication was supported from the research supported by an NIH/NINR funded program K23NR014107 (PI: W-TC), NIH/NIMHD R03MD012210 (PI: W-TC), (R25MH087217; PI: Kershaw, S. Trace), NIH/NIMH P30MH058107 (PI: Steven Shoptaw), and 1P30AI152501; PI-Jerome A. Zack, 2022 EHE Supplement PI: W-TC.

## Conflict of interest

The authors declare that the research was conducted in the absence of any commercial or financial relationships that could be construed as a potential conflict of interest.

## Publisher’s note

All claims expressed in this article are solely those of the authors and do not necessarily represent those of their affiliated organizations, or those of the publisher, the editors and the reviewers. Any product that may be evaluated in this article, or claim that may be made by its manufacturer, is not guaranteed or endorsed by the publisher.
